# MuscNet, a Weighted Voting Model of Multi-Source Connectivity Networks to Predict Mild Cognitive Impairment Using Resting-State Functional MRI

**DOI:** 10.1109/access.2020.3025828

**Published:** 2020-09-22

**Authors:** JIALIANG LI, ZHAOMIN YAO, MEIYU DUAN, SHUAI LIU, FEI LI, HAIYANG ZHU, ZHIQIANG XIA, LAN HUANG, FENGFENG ZHOU

**Affiliations:** 1 BioKnow Health Informatics Laboratory, College of Software, Jilin University, Changchun 130012, China; 2 Key Laboratory of Symbolic Computation and Knowledge Engineering of Ministry of Education, Jilin University, Changchun 130012, China; 3 BioKnow Health Informatics Laboratory, College of Computer Science and Technology, Jilin University, Changchun 130012, China; 4 Cancer Systems Biology Center, China-Japan Union Hospital of Jilin University, Changchun 130012, China

**Keywords:** Mild cognitive impairment, Alzheimer’s disease, resting-state functional MRI, brain functional connectivity network, multi-source connectivity network, weighted voting model, MuscNet

## Abstract

The neurological disorder mild cognitive impairment (MCI) demonstrates minor impacts on the patient’s daily activities and may be ignored as the status of normal aging. But some of the MCI patients may further develop into severe statuses like Alzheimer’s disease (AD). The brain functional connectivity network (BFCN) was usually constructed from the resting-state functional magnetic resonance imaging (rs-fMRI) data. This technology has been widely used to detect the neurodegenerative dementia and to reveal the intrinsic mechanism of neural activities. The BFCN edge was usually determined by the pairwise correlation between the brain regions. This study proposed a weighted voting model of multi-source connectivity networks (MuscNet) by integrating multiple BFCNs of different correlation coefficients. Our model was further improved by removing redundant features. The experimental data demonstrated that different BFCNs contributed complementary information to each other and MuscNet outperformed the existing models on detecting MCI patients. The previous study suggested the existence of multiple solutions with similarly good performance for a machine learning problem. The proposed model MuscNet utilized a weighted voting strategy to slightly outperform the existing studies, suggesting an effective way to fuse multiple base models. The reason may need further theoretical investigations about why different base models contribute to each other for the MCI prediction.

## I. INTRODUCTION

Alzheimer’s disease (AD) is one of the most dangerous and frequently-occurred brain diseases, and this irreversible neuro-degenerative dementia remains to have no effective treatments [1]. AD may develop from mild cognitive impairment (MCI), which has become the investigation focus of the AD researchers [2]. According to [3], the functionally alive brain may be modeled as a graph, with the functional brain regions (also called region of interest, or ROI) as nodes and significant correlations between nodes as edges, which are calculated using the time series data of bloodoxygenation-level-dependent (BOLD) signals of each ROI. Such a network was defined as the basal forebrain cholinergic neurons (BFCN) and was expected to reveal intrinsic mechanisms about BFCNs, etc[4]. The static Pearson correlation coefficient (PCC)-based BFCN has demonstrated promising predicting performances on the diagnosis of MCI, AD and a few other diseases [5]–[7]. The partial time series data of blood-oxygenation-level-dependent (BOLD) signals were used to represent the temporary graph nodes and to construct the dynamic BFCN [8], [9].

The above-mentioned network was regarded as the low-order BFCN and in [5], the high-order version BFCN (HON) was constructed by calculating weighted-graph local clustering coefficients as the features of brain regions. In [7], dynamic versions of low-order (LoM) and high-order BFCNs (HiO) were constructed simultaneously using Matrix Variate Normal Distribution (MVND), and a fusion model (FuMO) integrating both low-order and high-order BFCN was further proposed. Although the high order BFCN demonstrated better diagnosis performance on neurodegenerative dementia [10], researches in [11] indicated that the integration of both low-order and high-order BFCNs may complement each other and facilitate a better diagnosis result.

Brain is a comprehensive and dynamic network of ROIs and may be formulated by various versions of BFCNs through different definitions of the edges including HON [5], FuMO [7], sparse network [12], ICA [13], etc. Pearson correlation coefficient (PCC) was the most popular formulation of the ROI edges for its simplicity and effectiveness. Due to the PCC’s limitation on describing the non-linear correlation, various other inter-variable similarity metrics were introduced to define the edges, e.g. Kendall correlation coefficient (KCC) [14, 15], Spearman correlation coefficient (SCC) [16], and cosine similarity (CS) [17], etc. The correlation metrics maximal information coefficient (MIC) has been proven to have outstanding performance in capturing both linear and nonlinear relationships between two variables [18] and has been successfully applied to connectivity network construction [19] and schizophrenia disease detection [20].

Considering that brain functional network cannot be modeled simply using one correlation coefficient, integrated modeling of different BFCNs may improve the disease diagnosis performances. In [10], the correlation’s correlation of multiple levels of high-order BFCNs were calculated and the linear fusion of those BFCNs in different levels facilitated an important improvement in the computational diagnosis of Autism Spectrum Disorders. Relevant conclusion in [21] showed that a multi-task intrinsic network fusing within- and between-task interactions of patients and controls extracted by general linear model (GLM) and ICA can improve the schizophrenia diagnosis. However, those works only applied the traditional tool like PCC and ICA for the base model, which may be limited.

This study hypothesized that the sensitive correlation metrics MIC may capture hidden information from the noisy brain rs-fMRI data compared to other traditional coefficients, and the combination of MIC and other coefficients may improve the diagnosis. Inspired by the above works, we presented a new data fusion method considering the BFCNs constructed by various correlation coefficients as data source. To the best of our knowledge, this is the first work of utilizing MIC as the correlation metrics in the BFCN construction for MCI diagnosis and the first work of combining BFCNs with different correlation measuring metrics. Furthermore, considering the computational challenge of the large feature number of a BFCN, we explored the effects of different feature selection methods on the MCI prediction performance. Our experimental data demonstrated that the MCI diagnosis may be improved by integrating multiple correlation metrics and selecting appropriate features.

## II. MATERIALS AND METHODS

### A. DATASET SUMMARY

This study used the publicly available neuro-imaging dataset from the Alzheimer’s Disease Neuroimaging Initiative (ADNI) database [22]. ADNI is a longitudinal multi-center consortium aiming to accumulate and provide the clinical, imaging, genetics and biochemical datasets for the early detection and tracking of Alzheimer’s disease (AD). After a decade’s ongoing effort, the ADNI database released to the public researchers with the multiple types of neuro-imaging data and other clinical information. ADNI has served as a major data source for the researchers of detecting the onset and progression of mild cognitive impairment (MCI) [23], [24].

This study used the same dataset as in [7], including 68 mild cognitive impairment (MCI) patients and 69 Normal Controls (NC) from the ADNI database. The rs-fMRI of these participants were scanned by 3.0T Philips MRI scanner. The following scanner settings were utilized: TR/TE = 3000/30 mm, flip angle = 80^◦^, imaging matrix size = 64 * 64, 48 slices and 140 volumes, and slice thickness = 3.3mm. Each rs-fMRI image data was processed by the following well-accepted protocol using the software SPM8, which is available at https://www.fil.ion.ucl.ac.uk/spm/software/spm8/. In order to make a consistent comparison, the samples were selected using the same criteria as the study [7].

The first three volumes of each participant were discarded to ensure the magnetization equilibrium. The rest 137 volumes of each participant were then pre-processed with correction and normalization. The rs-fMRI imaging data was regressed to reduce the effects of nuisance signals including ventricle, white matter signals and six head-motion profiles. The Automated Anatomical Labeling (AAL) template atlas [25] was utilized to divide the generated Blood Oxygen Level Dependent (BOLD) time series signals into 116 regions of interest (ROIs). Finally, the mean rs-fMRI time series signals of each ROI (consisting of 137 volumes) were band-pass filtered from 0.01 to 0.08 Hz to generate the data matrix of each participant for constructing BFCN. The pre-processed data was provided by [7].

### B. CONSTRUCTION OF MuscNet

**FIGURE 1.**
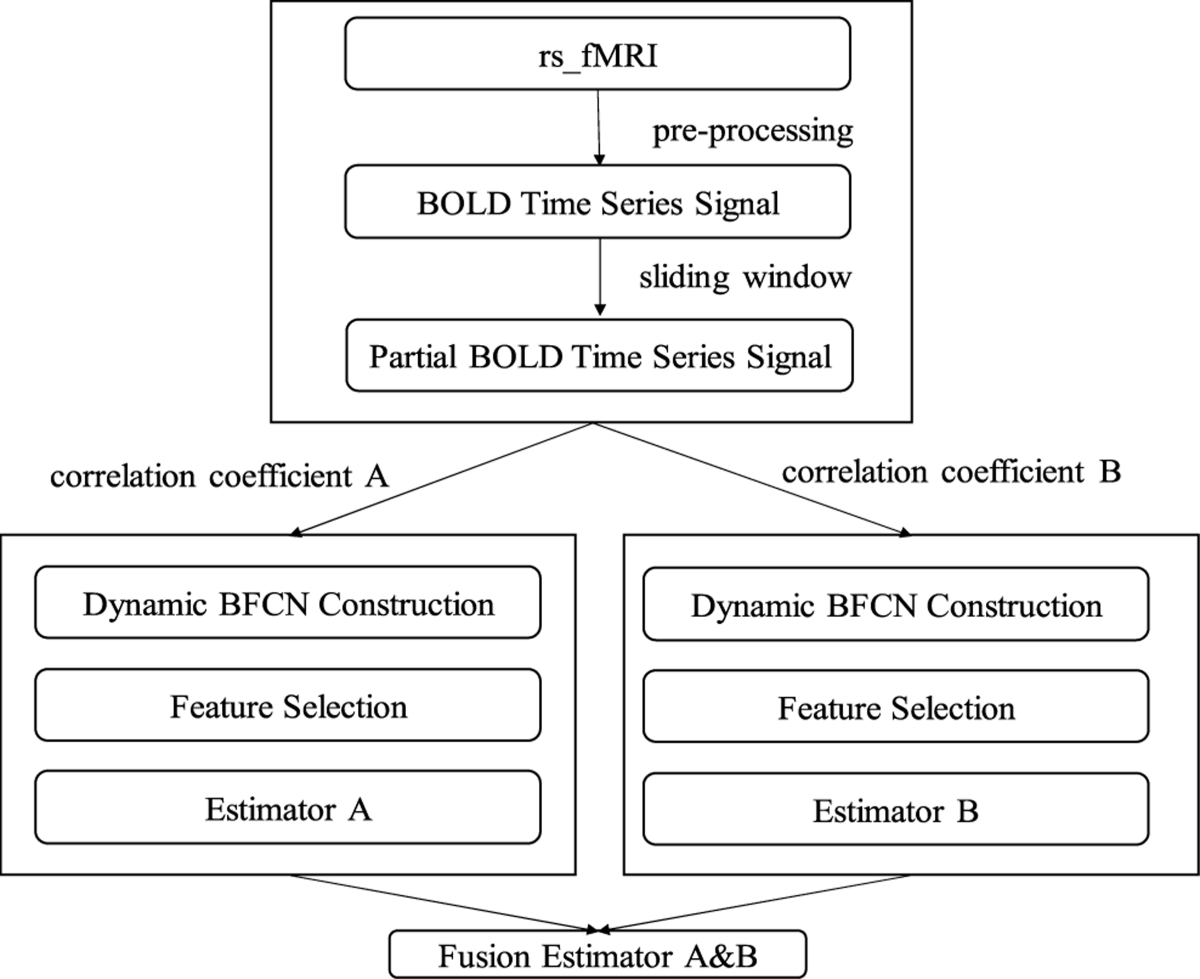
Multi-source dynamic BFCN structure. For each correlation coefficient, we can follow the above structure to construct the corresponding BFCN and to further integrate the pairwise estimators.

This study constructed a multi-source connectivity network (MuscNet) by integrating four classic metrics for measuring linear correlation coefficients (CC), including PCC, SCC, KCC and CS, and the sensitive metrics MIC, as illustrated in Figure 1. PCC was considered as equivalent to the decentralized CS. Both SCC and KCC were non-linear tolerant.

#### 1) STATIC BFCN

The correlation ${\text{W}}_{\text{ij}}^{\text{corr}}$ between ROI_i_ and ROI_j_ of subject n was defined as follows:

(1) $${\text{W}}_{\text{ij}}^{\text{corr}}=corr\left({\text{ROI}}_{\text{i}},{\text{ROI}}_{\text{j}}\right)$$

where corr() can be replaced by one specific correlation coefficient metrics. Taking PCC and MIC as examples, respectively. ${\text{W}}_{\text{ij}}^{\text{PCC}}$ may calculated as:

(2) $${\text{w}}_{\text{ij}}^{\text{PCC}}=\frac{\sum_{\text{m}=1}^{{\text{num}}_{\text{R}O1}}\left({\text{V}}_{\text{i}}^{\text{m}}-{\bar{V}}_{\text{i}}\right)\left({\text{V}}_{\text{j}}^{\text{m}}-{\bar{V}}_{\text{j}}\right)}{{\sqrt{\sum_{\text{m}=1}^{{\text{num}}_{\text{RO}}}\left({\text{V}}_{\text{i}}^{\text{m}}-{\bar{V}}_{\text{i}}\right)}}^{2}{\sqrt{\sum_{\text{m}=1}^{{\text{num}}_{\text{R}O1}}\left({\text{V}}_{\text{j}}^{\text{m}}-{\bar{V}}_{\text{j}}\right)}}^{2}}$$

where ${\text{V}}_{\text{i}}^{\text{m}}$ was defined as the m_th_ element in BOLD time series signal vector of ROI_i_ of subject n. And ${\text{W}}_{\text{ij}}^{\text{MIC}}$ may be calculated as:

(3) $${\text{W}}_{\text{ij}}^{\text{MIC}}=\frac{\text{I}\left({\text{V}}_{\text{i}},{\text{V}}_{\text{j}}\right)}{\min\left\{\text{H}\left({\text{V}}_{\text{i}}\right),\text{H}\left({\text{V}}_{\text{j}}\right)\right\}}$$

where I(V_i_,V_j_) was the mutual information between V_i_ and V_j_, and H(V_i_) was the Shannon information entropy of the variable V_i_. The calculation of the MIC and the Shannon information entropy were implemented as the function MINE() in the Python package minepy version 1.2.3 [19]. After pre-processing, we can obtain a data matrix with shape (num_subj_, num_volume_, num_ROI_), where num_subj_ = 137, num_volume_ = 137, num_ROI_ = 116, meant that each subject had 137 volumes, each volume included 116 BOLD signals corresponding to each ROI. We can also transpose it into (num_subj_, num_ROI_, num_volume_), which meant each ROI had a BOLD signal vector with length num_volume_. Then the basic correlation based BFCN can be constructed as follows: 1) Calculate pairwise correlation coefficient ${\text{W}}_{\text{ij}}^{\text{corr}\;}$ of all subjects. 2) Concatenate all ${\text{W}}_{\text{ij}}^{\text{corr}\;}$ of one subject into a square matrix with shape (num_ROI_, num_ROI_), which was called BFCN correlation square. 3) Flatten the BFCN correlation square, and get the BFCN correlation feature vector F_n_ of each subject with length (num_ROI_ * num_ROI_). Supplementary Figure S1 illustrated the BFCN correlation matrices based on PCC and MIC. As for the BFCN correlation matrices based on other correlation coefficients, corresponding illustration can be found in Supplementary Figure S2 due to the page limit.

#### 2) DYNAMIC BFCN

This study extracted the following number of sliding-window segments from the whole time series signal:

(4) $${\;\text{num}\;}_{\text{window}\;}=\left(\frac{{\;\text{num}\;}_{\text{volume}\;}-{\;\text{window}\;}_{\text{size}\;}}{{\;\text{window}\;}_{\text{step}\;}}+1\right)$$

where window_size_ is the length of each window, and window_step_ is the distance between two successive windows. For example, if window_size_ = 50, window_step_ = 8, the 137 volumes may be converted to 11 segments to construct 11 temporal BFCNs. Following the same procedure as in static BFCN construction, we can produce num_window_ temporal BFCN correlation squares. Then the average of these num_window_ temporal correlation squares will be flattened as the dynamic BFCN correlation feature vector DF_n_ of each subject. As similar to [7], window_size_ was set to (50, 70, 90, 110), while the range of window_step_ was (1, 2, 4, 8, 10), respectively.

#### 3) MUSCNET

Inspired by the previous works [7], [10], [21] using data fusion to improve the model performance, we constructed MuscNet by integrating multiple estimators trained by dynamic BFCNs based on different correlation coefficient metrics. The whole construction was as follows: 1) Divide the whole time series signals into several segments by sliding-window. 2) For each segment, get the corresponding BFCN correlation feature vector F_n_ of each subject. 3)Train the estimator group by F_n_s corresponding to various correlation coefficients. 4) Fuse the estimator pairwise. Specifically, the decision scores from each pair of estimators generated from the above process were linearly fused by a weighting parameter *α*, which ranges from 0.1 to 0.9 with step 0.1. So MuscNet is a weighted voting strategy to fusing multi-source connectivity networks. For illustration purposes, each MuscNet may be described with the two correlation coefficient metrics. For example, “PCC&MCC” represented the MuscNet combining the estimator using PCC with the estimator using MCC.

### C. FEATURE SELECTION METHODS

This study utilized three popular filter algorithms to select features for the binary classification problem of MCI and NC samples. Ttest is the most popular feature selection algorithm [26] and has demonstrated its effectiveness on the AD diagnosis [7]. Wilcoxon rank-sum Test (Wtest) [27], also called Wilcoxon-Mann-Whitney Test, is considered as an alternative to T-test for its increased robustness [28]. Kolmogorov-Smirnov Test (KStest) is usually employed to estimate how much two distributions are related to each other and its promising performance for eliminating redundant features has been proved in [29]. Besides, we further discussed the impact of different filter thresholds for the statistical Pvalues on classification performance.

### D. CLASSIFICATION PERFORMANCE

#### EVALUATION METRICS

For a fair comparison with the existing studies, we employed the leave-one-out cross validation (LOOCV) to measure the classification performances of MCI and NC samples. To compare the classification performance of different correlation coefficients combined with different feature selection methods and different parameters, we set one LOOCV for each combination. Specifically, each LOOCV trained one model on (num_subj_−1) subjects and predicted the class label of the rest one sample. Performance of each combination can be gained after num_subj_ repeats of LOOCV. To further verify the performance of the best combination, we adopted the two-level LOOCV, which is also called nested-LOOCV.

This study evaluated a classification model using three popular classification performance metrics, i.e., Accuracy (Acc), Sensitivity (Sn) and Specificity (Sp). The MCI and NC participants were regarded as positive and negative samples, respectively. The total numbers of positive and correctly predicted positive samples were defined as P and TP (true positive). FN = P - TP was the number of those incorrectly predicted positive samples. The numbers of correctly and incorrectly predicted negative samples were TN (true negative) and FP (false positive), respectively. And there were N = TN + FP negative samples. The performance metrics Sn and Sp were defined as the percentages of correctly predicted positive and negative samples, respectively. That is to say, Sn = TP/(TP + FN) = TP/P and Sp = TN/(TN + FP) = TN/N. And Acc was the overall percentage of correctly predicted samples, i.e., Acc = (TP + TN)/(P + N).

For each test sample of an LOOCV, we can get a series of selected feature subsets. Further analysis on these feature subsets was expected to bring inspiration to the diagnosis of MCI.

## III. RESULTS AND DISCUSSION

### A. EVALUATION OF BFCNs USING DIFFERENT CORRELATION COEFFICIENT METRICS

**FIGURE 2.**
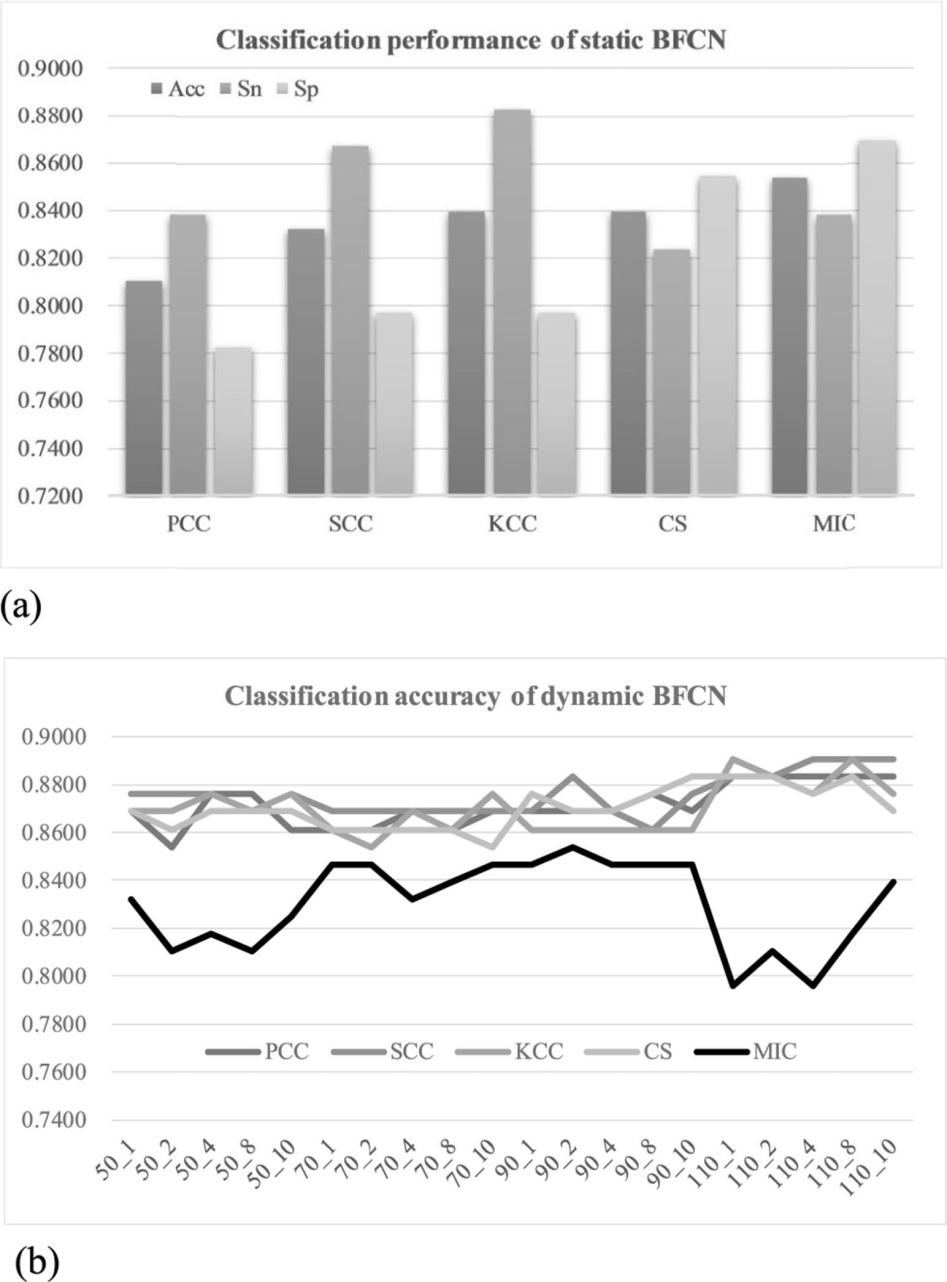
Classification performance of BFCNs based on different parameters. (a) The static BFNCs using different correlation coefficient metrics. (b) The dynamic BFCNs using different window_size_ and window_step_ for different correlation coefficient metrics. The horizontal axis was in the format of window_size__window_step_ and the vertical axis was the classification accuracy. Features with Ttest Pvalue < 0.05 was chosen to calculate the classification performances for both sub-figures.

This study firstly evaluated how different correlation coefficient metrics impact the binary classification performances of the investigated problem, as shown in Figure 2. The support vector machine with the linear kernel (lSVM) was used as the classifier for the evaluation [30]. The metrics MIC achieved the best classification accuracy (Acc) for the static BFCN-based models, as shown in Figure 2 (a). The MIC-based static BFCN also achieved a less-biased pair of Sn and Sp. The two metrics SCC/KCC performed better in Sn than MIC, but their Sp values were much smaller than that of MIC. But for the dynamic BFCNs, MIC performed worse than all the other four correlation coefficient metrics, as shown in Figure 2 (b). Further detailed comparison in Sn and Sp can be found in Supplementary Figure S3. The experimental data suggested that there was no single correlation coefficient metrics performing the best for both static and dynamic BFCN-based MCI classifications.

### B. EVALUATION OF MuscNet

**FIGURE 3.**
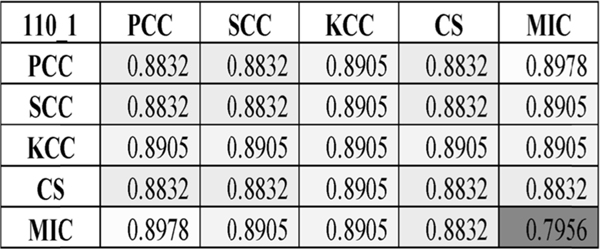
MuscNet classification accuracies based on dynamic BFCNs of one CC or a CC duet. The parameter “110_1” in the top left corner represented the two parameters window_size_ = 110 and window_step_ = 1. As correlation heatmap, this comparison heatmap was also diagonally symmetrical. The diagonal represented a dynamic BFCN based on single correlation coefficient (CC), and the grids represented the integrated dynamic BFCNs of a CC duet. The heatmap background color was lighter if the value was larger. Features with Ttest Pvalue < 0.05 were chosen to calculate the classification performances.

We then evaluated MuscNet models with different correlation coefficient (CC) duets. As shown in Figure 3 and Supplementary Figure S4, MuscNet significantly outperformed dynamic BFCN based on one correlation coefficient with a maximal accuracy 0.8978. Except for the KCC-based BFCNs, all the other CC-based BFCNs may be improved by integrating one more CC metrics. MIC performed the worst if it was used alone to construct the dynamic BFCN (Acc = 0.7956) but improved both PCC- and SCC-based models if the MIC was integrated with one of these two CC metrics. And MIC didn’t decrease the classification accuracies of the other two CC-based models if being integrated with KCC- and CS-based models. So MIC may be a good complementary CC metrics to be integrated with the other CC metrics.

### C. EVALUATION OF FEATURE SELECTION ALGORITHMS

**FIGURE 4.**
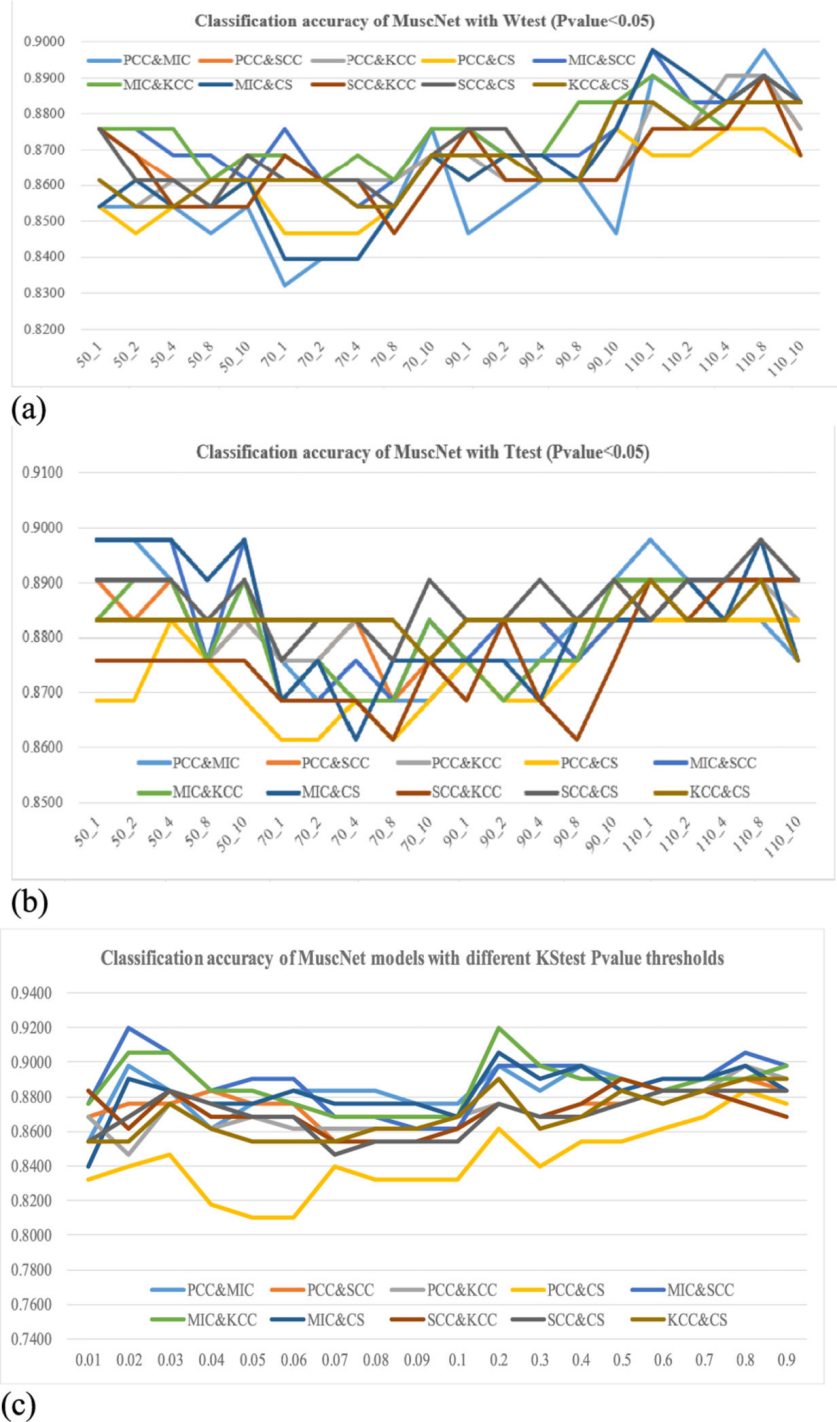
Classification accuracy of MuscNet with different feature selection methods (p-value = 0.05). The notation “PCC&MIC” represented the MuscNet model integrating the PCC- and MIC-based dynamic BFCNs. The horizontal axis was the parameter duet window_size__window_step_. The vertical axis was the classification accuracy calculated using the features with the filter Pvalue < 0.05, where the filter algorithm could be (a) Wtest, (b) Ttest and (c) KStest.

Different filter algorithms were evaluated for their improvements on the MuscNet models, as shown in Figure 4. The above-discussed results were carried out using Ttest as the feature selection algorithm. Ttest may not always perform the best on different datasets [31], [32]. So we further evaluated the three filter algorithms Wtest, Ttest and KStest. Features were selected for training the classification model if their filter-based Pvalues were smaller than 0.05. Firstly, Ttest didn’t always achieve the best classification performances, as shown in Figure 4 (a)–(c). Ttest and Wtest performed similarly well on the classification accuracies, and KStest achieved the best performances (Acc = 0.9124, Sn = 0.8971 and Sp = 0.9275) with two parameters window_size_ = 90 and window_step_ = 8 and the integration of MIC and KCC.

**FIGURE 5.**
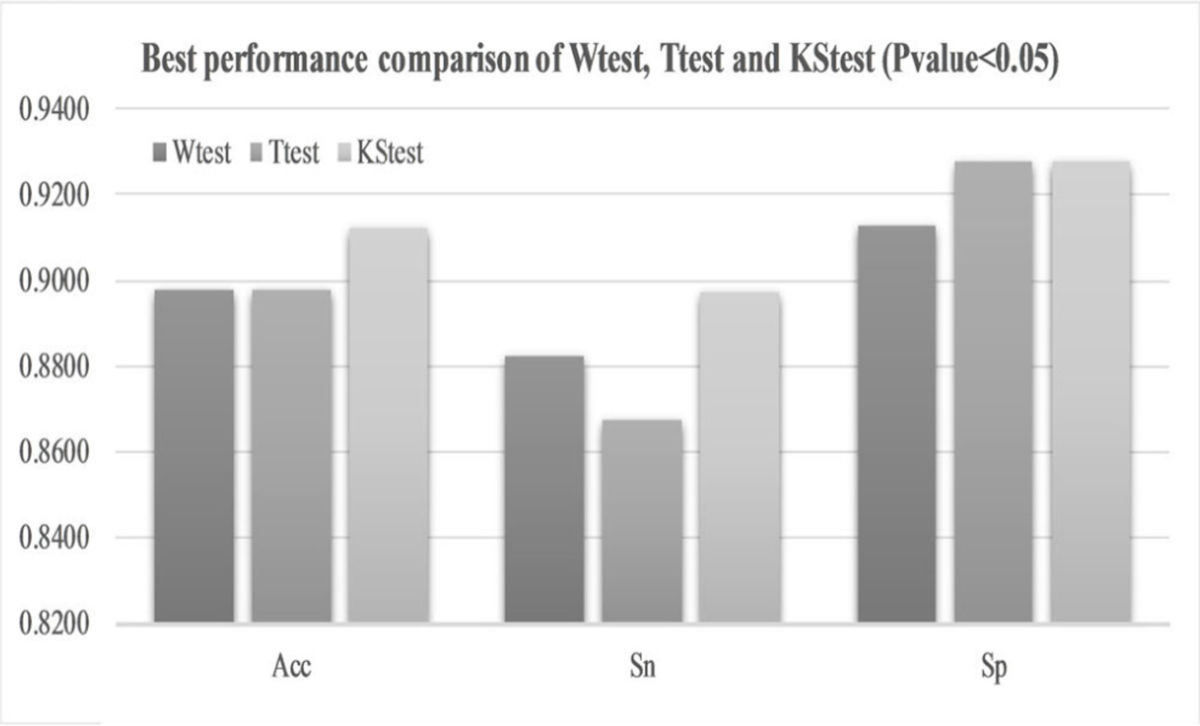
Best performance comparison among Wtest, Ttest and KStest (Pvalue < 0.05). The horizontal axis was the three classification performance metrics, Acc, Sn and Sp. The horizontal axis was the three classification performance metrics of different filter algorithms, respectively. The vertical axis was the corresponding values of each metric.

Figure 4 supported the above observation that MIC served as a good source of complementary information to the other CC metrics, although MIC didn’t perform well by itself. The best model with Wtest was integrated from PCC- and MIC-based dynamic BFCNs using the two parameters window_size_ = 110 and window_step_ = 8. The best model with Ttest was integrated from PCC- and MIC-based dynamic BFCNs with window_size_ = 50, window_step_ = 1 as sliding window parameters. And the best model with KStest was integrated from MIC- and KCC-based dynamic BFCNs with window_size_ = 90, window_step_ = 8, as shown in Figure 5.

### D. PROMOTION BY PARAMETERS TUNING

**FIGURE 6.**
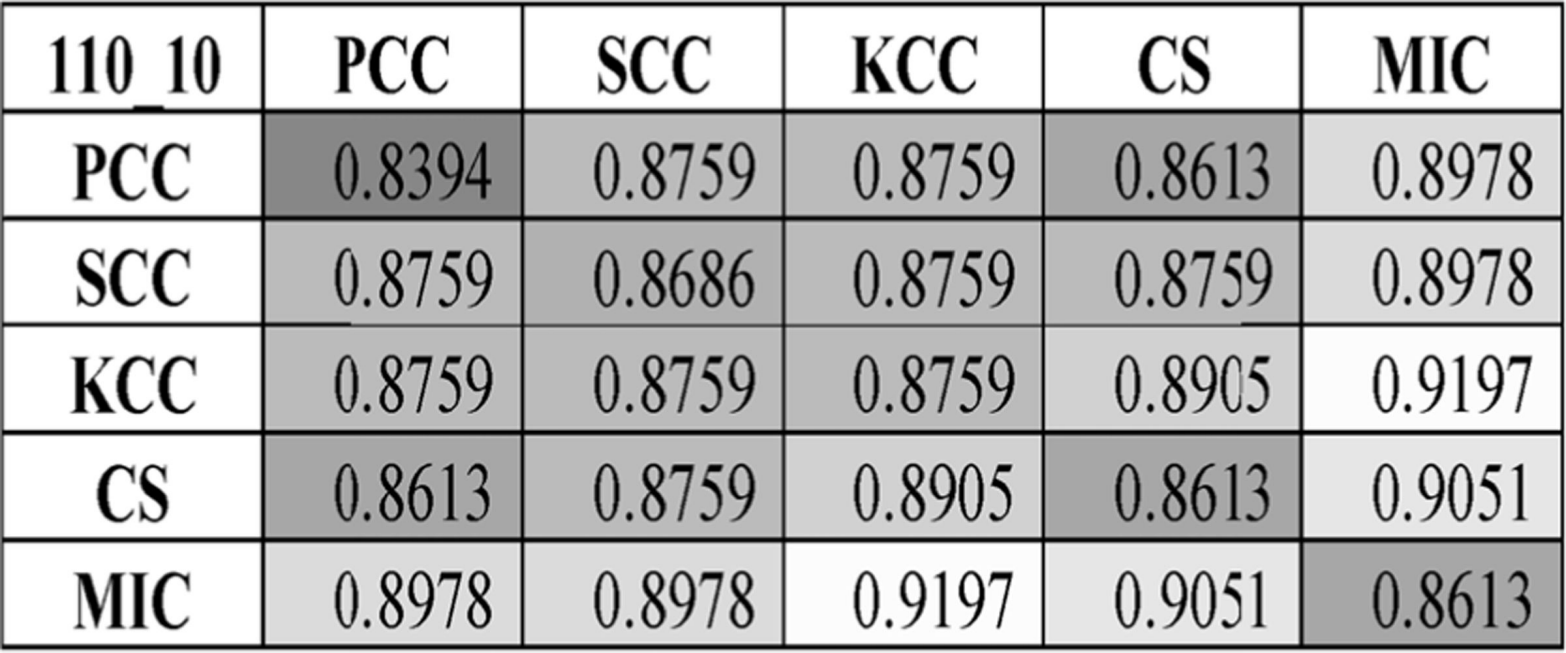
The classification accuracy heatmap. This heatmap showed the comparison between dynamic BFCNs based on one CC and integrated CC duets. Features with the KStest (Pvalue < 0.2) were selected for training the model. And the two parameters window_size_ = 110, window_step_ = 10 were set for the sliding windows. The heatmap background color was lighter if the value was larger.

We further investigated how the filter Pvalue threshold impacted the model prediction performances. Figure 6 illustrated that KStest-selected features with the Pvalue threshold 0.2 achieved the best prediction accuracy 0.9197 by integrating MIC- and KCC-based dynamic BFCNs. Results of each sliding window parameter combination can be found in Supplementary Figure S5. A similar pattern was also observed that MIC-based BFCN improved all the four other CC-based dynamic BFCNs.

**FIGURE 7.**
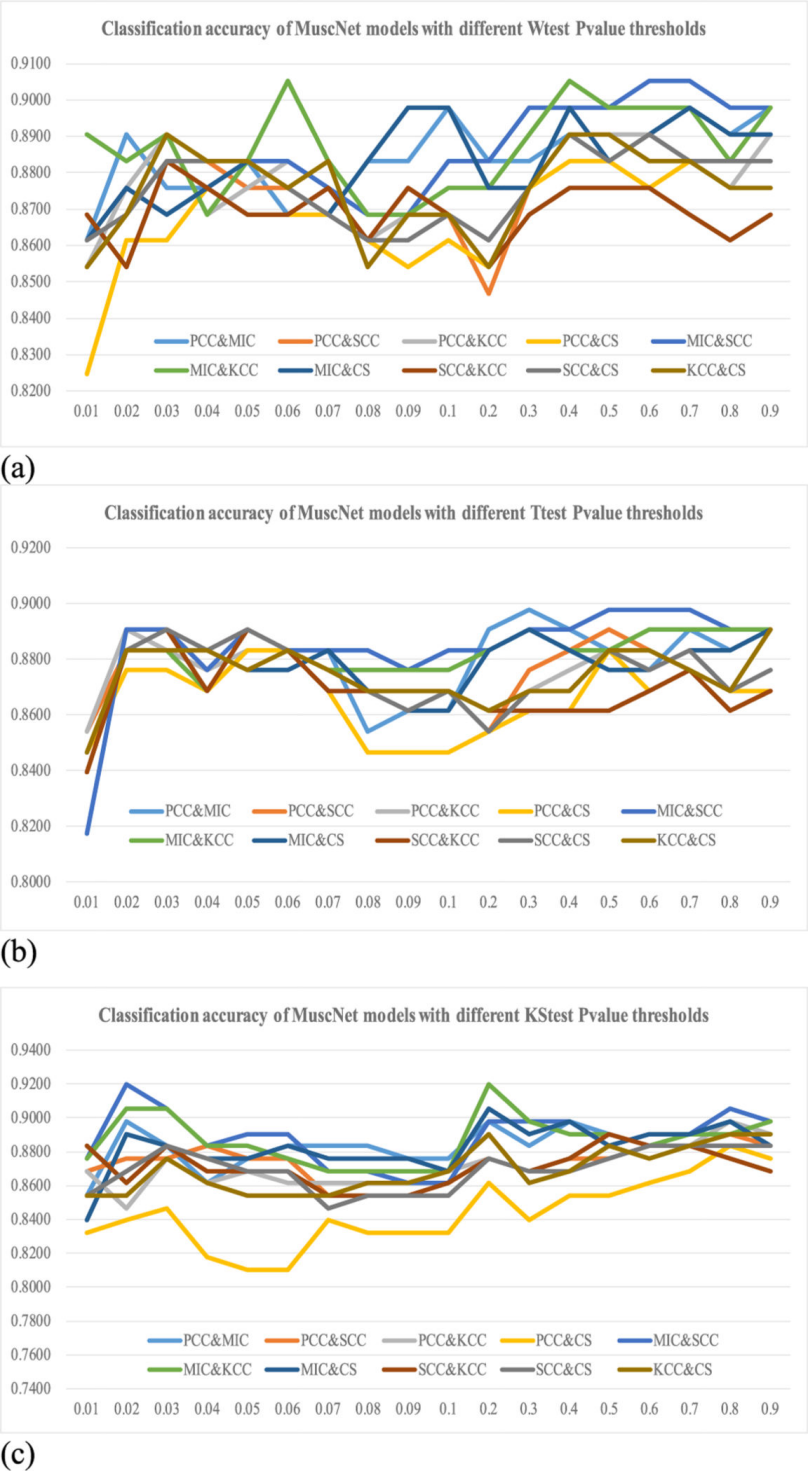
Classification accuracies of MuscNet models with different Pvalue thresholds. The horizontal axis was the filter Pvalue thresholds and the vertical axis was the classification accuracies. The evaluation was carried out for (a) Wtest, (b) Ttest and (c) KStest.

**FIGURE 8.**
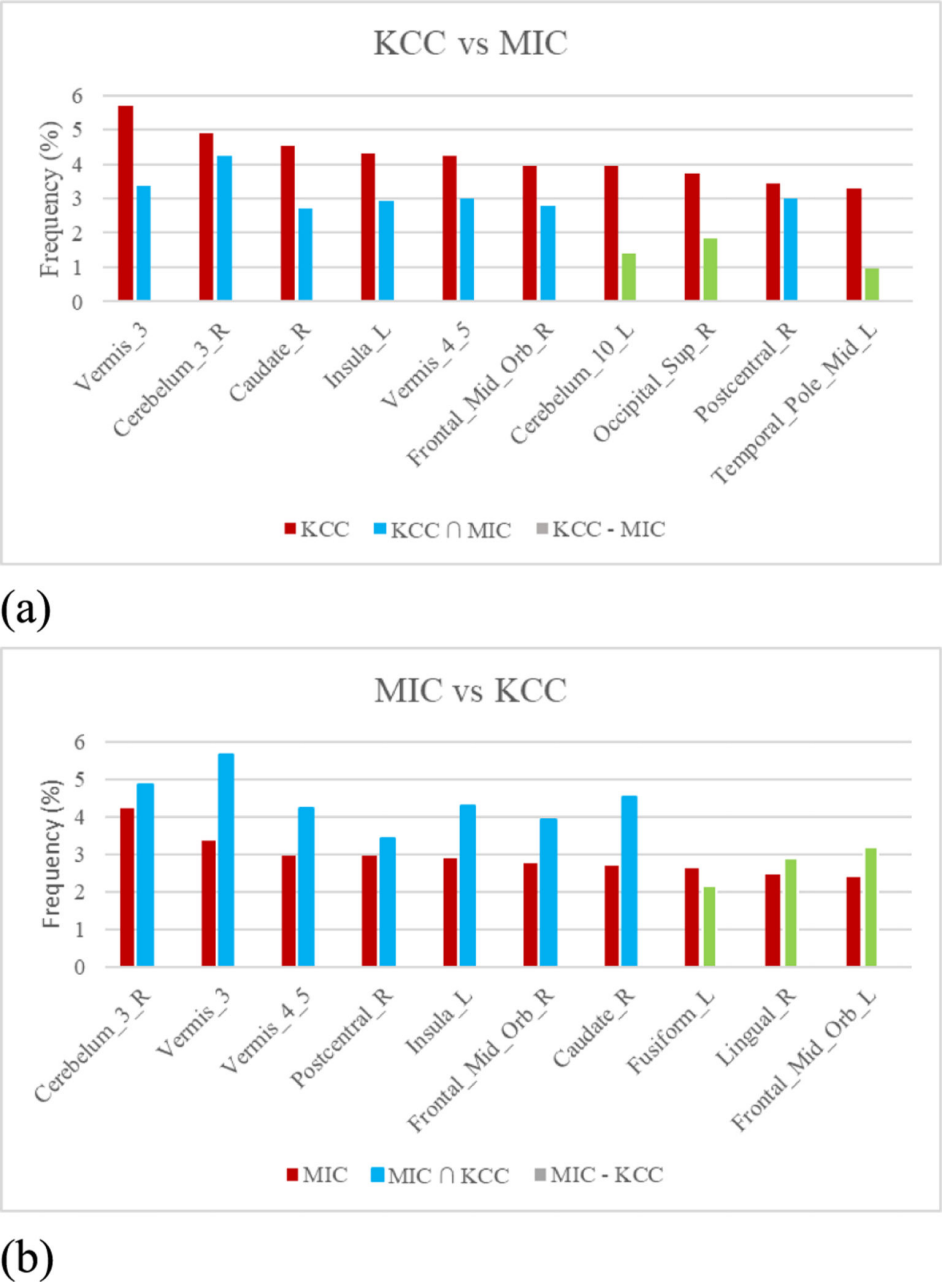
Comparison between top-10 features most frequently selected by MIC and KCC models. Column in red represents the top-10 features selected by one model, column in blue represents this feature also belongs to the top-10 features selected by the other model, and column in green represents this feature doesn’t belong to the top-10 features selected by the other model. The vertical axis of each figure was the selected frequency of one feature.

Figure 7 suggested that Ttest didn’t perform well on the dataset and the Ttest-based dynamic BFCN model only achieved Acc = 0.8978, as shown in Figure 8 (b). Wtest achieved a better overall accuracy 0.9051 by integrating the MIC- and SCC-based dynamic BFCNs. And the KStest achieved an even better accuracy 0.9197 for two models MIC&SCC and MIC&KCC. Verification results of this best model using the two-level LOOCV also get the accuracy 0.9197, suggesting that the set of the best parameters is also effective on the cross validation strategy.

### E. COMPARISON BETWEEN MuscNet AND THE EXISTING STUDIES

The MuscNet models were compared with the existing studies on the predicting performances of MCI patients, as shown in Table 1. To be specific, for HON method, window_size_ = 110, window_step_ = 1; for LoM, HiO and FuMO methods, window_size_ = 50, window_step_ = 8.

It is demonstrated in [5] that the high-order brain functional connectivity network (HON) performed effectively on detecting the MCI patients. Due to the variations in the samples used in each study, validation work in [7] demonstrated that the HON model achieved the accuracy 0.8207 on their dataset, as shown in Table 1. They proposed low-order model (LoM), high-order model (HiO) and the integrated fusion of both models (FuMO) to further improve the HON model, as shown in Table 1. The experimental data suggested that LoM achieved the best accuracy 0.9051 in their study.

The MuscNet model outperformed all these four models in the prediction performance metrics Acc and Sn, and achieved the same specificity (Sp = 0.9130) as the fusion model FuMO.

### F. BRAIN ROIs ASSOCIATED WITH THE MCI PREDICTION

As mentioned above, the best model in this study was the MIC&KCC model with window_size_ = 110, window_step_ = 10 and KStest Pvalue threshold 0.2. The top-10 most frequently selected ROIs of the best model were collected and analyzed for their known associations with MCI, as shown in Figure 8. A set of ROIs was selected during each iteration of the LOOCV, and the ROIs were ranked by their frequencies of being selected by these iterations. We can see from Figure 8 that the two model had more than half features in common, which means that the two models have a certain similarity in the description of the correlation, and they can both capture the core characteristics of rs-fMRI data. As for the difference set, all top-10 features selected by MIC also had a high selected frequency in the KCC model, not vice-versa.

Seven brain ROIs were frequently selected by both MIC and KCC models. Cerebellum was a biomarker ROI that contributed to the best MCI prediction accuracy, and it has already been observed to be involved in the development of both MCI and AD [33], [34]. Insula was a crucial structure for high-level cognition and was also observed in close associations with various neurodegenerative diseases [35], [36]. Other ROIs like lingual gyrus [37] and postcentral cingulate cortex [38] demonstrated to have reduced metabolism levels and were observed in the MCI or AD patients.

The previous study suggested the existence of multiple solutions with the similarly good performance for a machine learning problem [39], [40]. Difference correlation coefficients evaluated the MCI prediction problem from different perspectives. These correlation coefficients may generate different sets of features for the prediction models. And MuscNet achieved slightly better performance using different features than the other models, as shown in Table 1.

## IV. CONCLUSION

This study proposed the weighted voting-based fusion of a multi-source connectivity network (MuscNet) by integrating dynamic BFCNs using different correlation coefficients. This idea was inspired by the observation that the integration of low-order and high-order BFCN generated satisfying disease prediction performances.

Our experimental data demonstrated that the CC metrics MIC served as a complementary information source. Although MIC-based dynamic BFCN didn’t perform well, the integration of the MIC-based dynamic BFCN with other models usually improved these models. The proposed MuscNet model also improved the existing studies in predicting MCI. The statistical KStest performed the best on selecting features and the best model was the MIC&KCC-based dynamic BFCN.

## Supplementary Material

supp1-3025828

## Figures and Tables

**TABLE 1.** Comparison on MCI prediction performances with the existing studies. The performance metrics were accuracy (Acc), sensitivity (Sn) and specificity (Sp). The models HON, LoM, HiO, and FuMO were from the existing studies. And the MuscNet model was proposed in this study.

Method	Acc	Sn	Sp
HON	0.8207	0.8194	0.8377
LoM	0.9051	0.9118	0.8986
HiO	0.8394	0.8235	0.8551
FuMO	0.8905	0.8676	0.9130
**MuscNet**	**0.9197**	**0.9265**	**0.9130**
