# Supplementary material for: MuscNet, a Weighted Voting Model of Multi-Source Connectivity Networks to Predict Mild Cognitive Impairment Using Resting-State Functional MRI
Source: IEEE Access. Author manuscript; Available in PMC 2022 May 10. (PMC9090182; doi:10.1109/access.2020.3025828)
Supplement: supp1-3025828 [file NIHMS1633554-supplement-supp1-3025828.docx]

# MuscNet, a weighted voting model of multi-source connectivity networks to predict mild cognitive impairment using resting-state functional MRI

Jialiang Li^1^, Zhaomin Yao^3,2^, Meiyu Duan^2^, Shuai Liu^2^, Fei Li^1^, Haiyang Zhu^2^, Zhiqiang Xia^2^, Lan Huang^2^, Fengfeng Zhou^1, 2, #^, and the Alzheimer’s Disease Neuroimaging Initiative^*^

1. BioKnow Health Informatics Lab, College of Software, and Key Laboratory of Symbolic Computation and Knowledge Engineering of Ministry of Education, Jilin University, Changchun, Jilin, China, 130012.

2. BioKnow Health Informatics Lab, College of Computer Science and Technology, and Key Laboratory of Symbolic Computation and Knowledge Engineering of Ministry of Education, Jilin University, Changchun, Jilin, China, 130012.

3 Cancer Systems Biology Center, China-Japan Union Hospital of Jilin University, Changchun 130012, China.

# Correspondence may be addressed to Fengfeng Zhou: FengfengZhou@gmail.com or ffzhou@jlu.edu.cn . Lab web site: http://www.healthinformaticslab.org/ . Phone: +86-431-8516-6024. Fax: +86-431-8516-6024.

* Data used in preparation of this article were obtained from the Alzheimer’s Disease Neuroimaging Initiative (ADNI) database (adni.loni.usc.edu). As such, the investigators within the ADNI contributed to the design and implementation of ADNI and/or provided data but did not participate in analysis or writing of this report. A complete listing of ADNI investigators can be found at: http://adni.loni.usc.edu/wp-content/uploads/how_to_apply/ADNI_Acknowledgement_List.pdf

# Supplementary Figure S1

Pairwise correlation relationship of 116 ROIs based on different correlation coefficients. Specifically, (a) (b) (c) (d) showed the mean pairwise PCC relationship of MCI subjects, NC subjects and the pairwise PCC relationship of one MCI subject and NC subject, respectively. As a comparison, (e) (f) (g) (h) reflected the corresponding relationship based on nonlinear-suitable MIC.


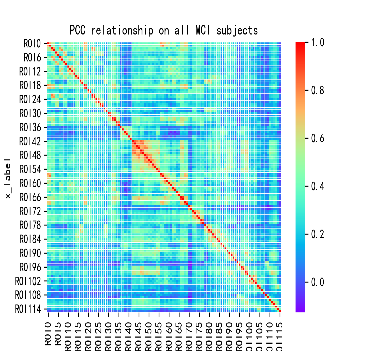

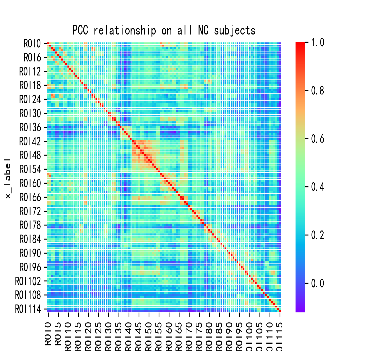


(a) (b)


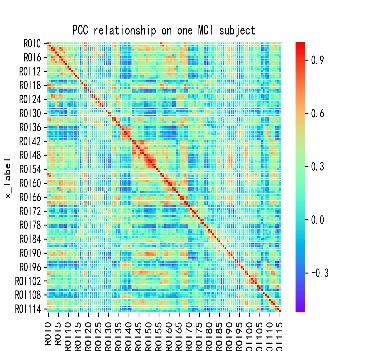

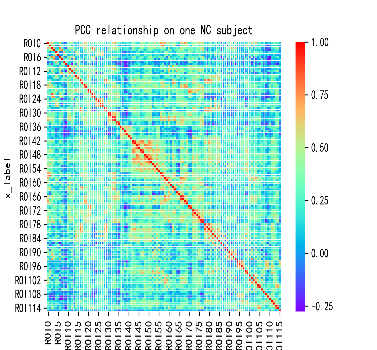


(c) (d)


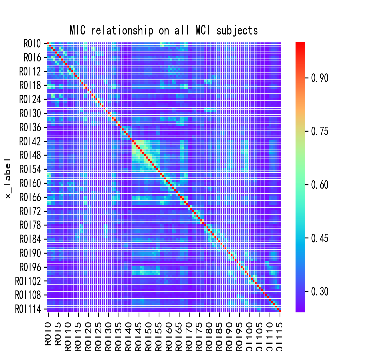

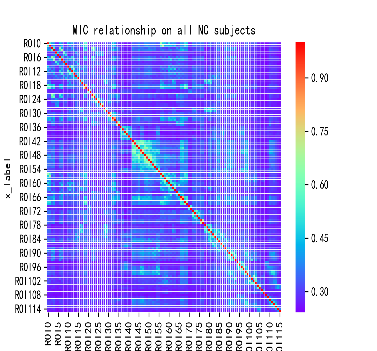


(e) (f)


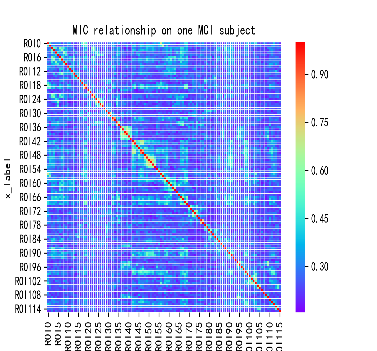

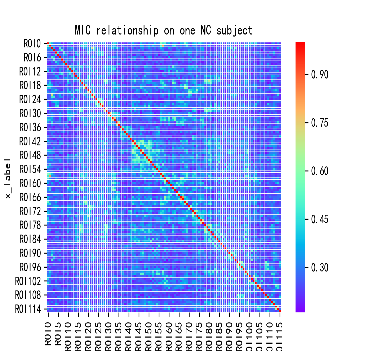


(g) (h)

# Supplementary Figure S2

The BFCN correlation matrices based on SCC, KCC and CS. Specifically, (a) (b) (c) (d) showed the mean pairwise SCC relationship of MCI subjects, NC subjects and the pairwise SCC relationship of one MCI subject and NC subject, respectively. As a comparison, (e) (f) (g) (h) and (i) (j) (k) (l) reflected the corresponding relationship based on KCC and CS, respectively.


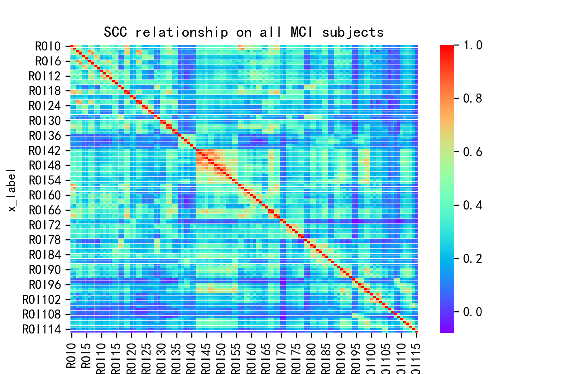

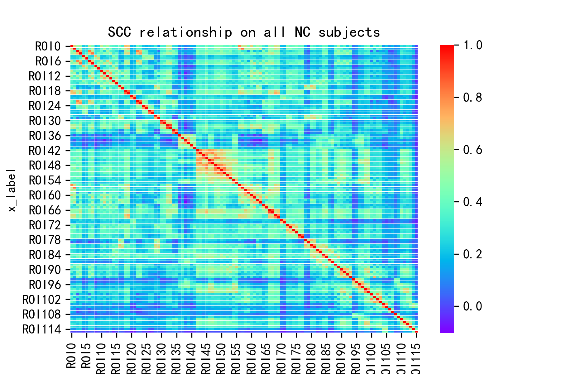


(a) (b)


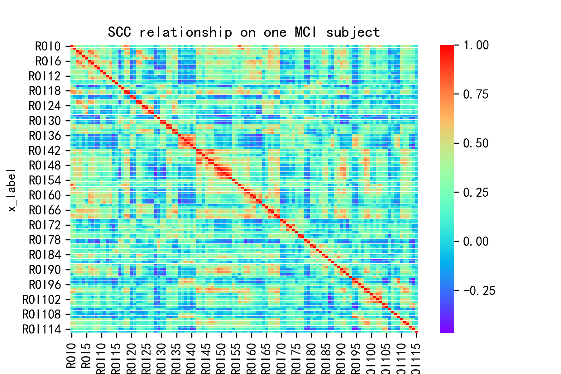

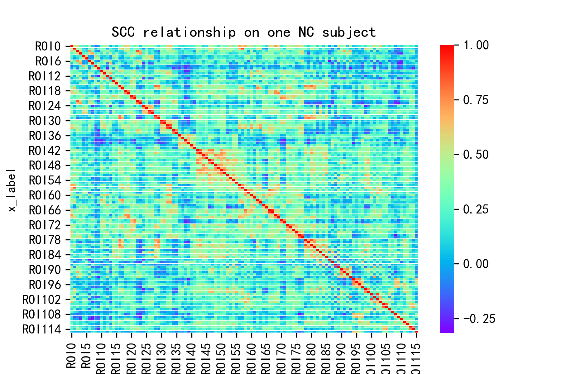


(c) (d)


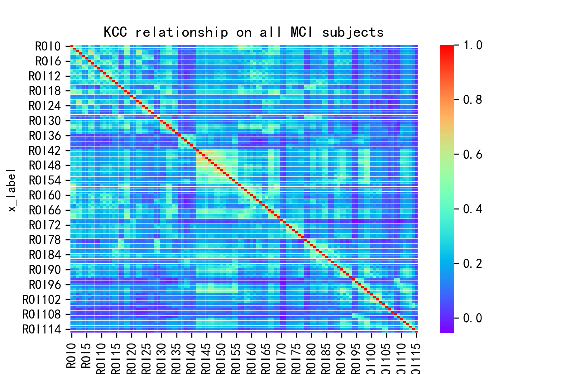

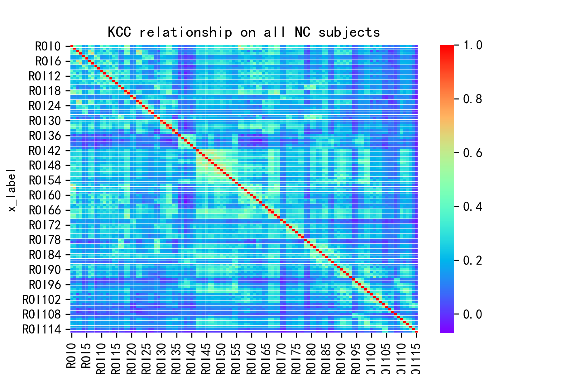


(e) (f)


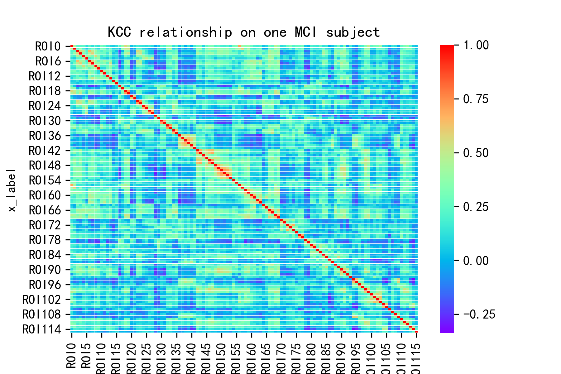

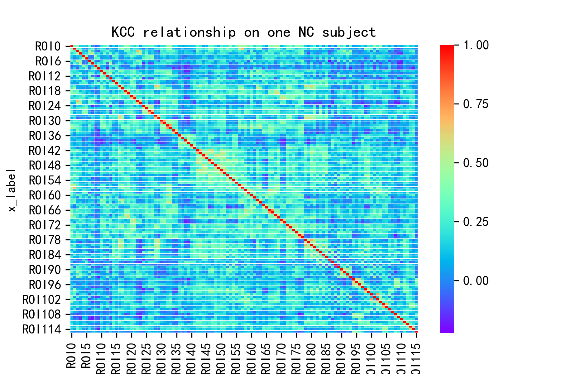


(g) (h)


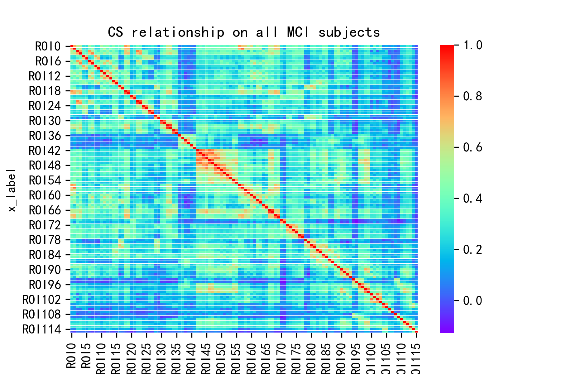

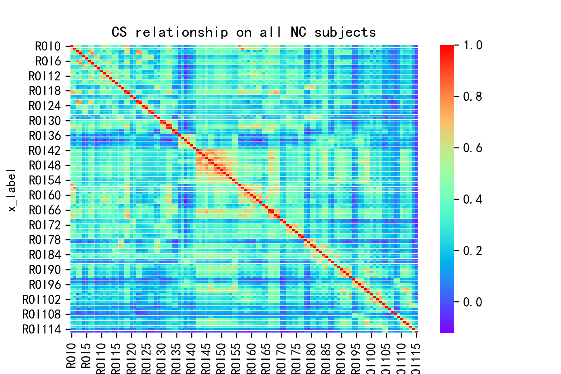


(i) (j)


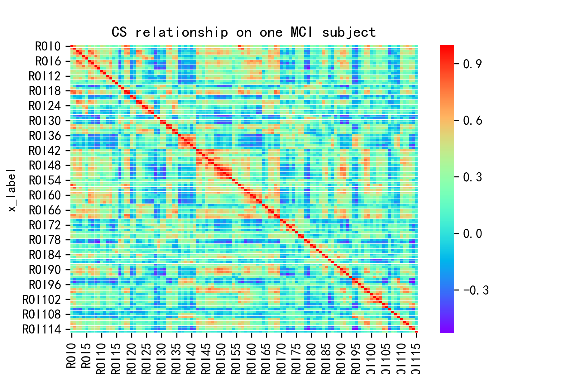

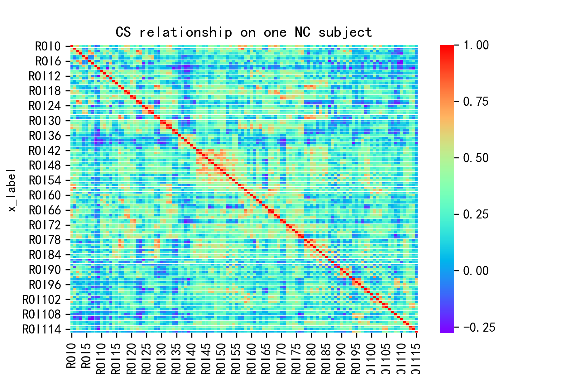


(k) (l)

# Supplementary Figure S3

Sensitivity and specificity of the dynamic BFCNs using different window_size_ and window_step_ for different correlation coefficient metrics. The horizontal axis was in the format of window_size__window_step_ and the vertical axis was the classification sensitivity (a) and specificity (b), respectively. Features with Ttest Pvalue<0.05 was chosen to calculate the classification performances for both sub-figures.


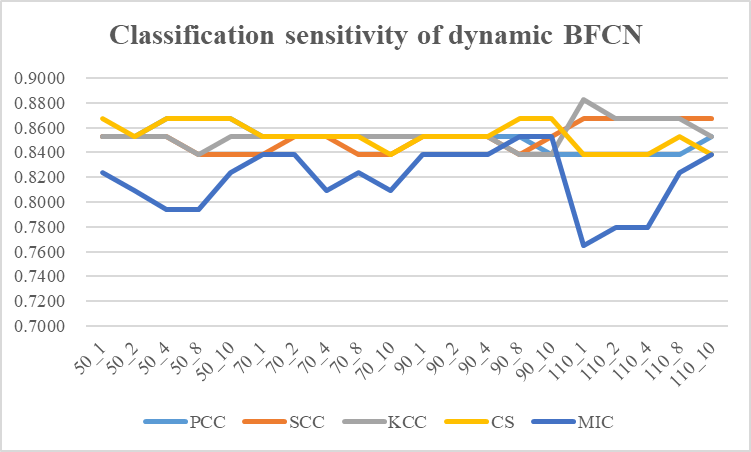


(a)


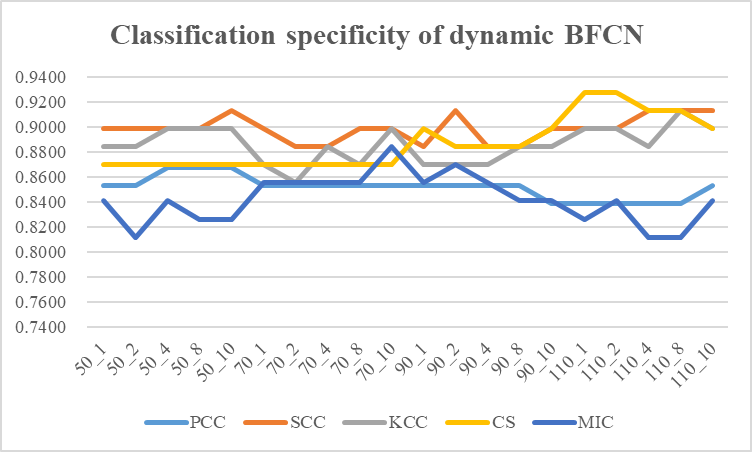


(b)

# Supplementary Figure S4

We employed comparison heatmap to illustrate the differences between MuscNet based on dynamic BFCNs of one CC and a CC duet. The parameter in the top left corner represented the two parameters $\text{window}_{\text{size}}$ and $\text{window}_{\text{step}\text{ }}(e.g. “50\_1” represented \text{window}_{\text{size}}=50$ and $\text{window}_{\text{step}}=1$). As correlation heatmap, this comparison heatmap was also diagonally symmetrical. The diagonal represented a dynamic BFCN based on single correlation coefficient (CC), and the grids represented the integrated dynamic BFCNs of a CC duet. The heatmap background color was lighter if the value was larger.

(a) (b)

(c) (d)

(e) (f)

(g) (h)

(i) (j)

(k) (l)

(m) (n)

(o) (p)

(q) (r)

(s) (t)

# Supplementary Figure S5

By employing KStest (Pvalue<0.2) as feature selection method, we further improved the classification accuracy of our MuscNet up to 0.9197. The best ACC was obtained by integrating MIC- and KCC-based dynamic BFCNs with $\text{window}_{\text{size}}\text{=110}$, $\text{window}_{\text{step}}\text{=10}$. Specific results of each sliding window parameter combination were showed as follows. The heatmap background color was lighter if the value was larger.

(a) (b)

(c) (d)

(e) (f)

(g) (h)

(i) (j)

(k) (l)

(m) (n)

(o) (p)

(q) (r)

(s) (t)
